# Impact of lifestyle factors and dietary patterns on serum uric acid levels and disease activity in gout: a systematic review

**DOI:** 10.1186/s41043-025-00982-4

**Published:** 2025-07-02

**Authors:** Mohammad Mustafa, Shahad Alshamrani, Lama Alghamdi, Hala Danish, Dana Alamoudi, Ghala Alshamrani, Abdullah Alagha, Adnan Alshaikh, Saher Alqarni, Yasser Bawazir

**Affiliations:** 1 Department of Medicine, University of Jeddah, Jeddah, Saudi Arabia; 2 Medical Student, College of Medicine, University of Jeddah, Jeddah, Saudi Arabia; 3 Medical Student, College of Medicine, King Saud Bin Abdulaziz for Health Sciences, Jeddah, Saudi Arabia; 4 Medical Intern, College of Medicine, King Abdulaziz University, Jeddah, Saudi Arabia; 5 Medical Student, College of Medicine, King Abdulaziz University, Jeddah, Saudi Arabia; 6 Medical department, King Abdulaziz University, Jeddah, KSA Saudi Arabia

**Keywords:** Arthritis, Gout, Dietary pattern, Lifestyle factor, Physical activity, Uric acids cohort study, Prospective, Synovial membranes, MNC infiltration

## Abstract

**Background:**

Gout is a common type of inflammatory arthritis caused by monosodium urate (MSU) crystal deposition in the joints. This leads to pain, swelling, and restricted motion. Although pharmacological treatments are effective, lifestyle and dietary factors play crucial roles in managing gout and its flares.

**Aim:**

This systematic review aimed to assess the effect of lifestyle factors, physical activity, and dietary patterns on serum uric acid levels and gout activity.

**Methods:**

A search of PubMed, BMJ journals, and Google Scholar identified eight studies (five prospective cohort studies, two case-cross-over studies, and one randomized controlled trial), involving 47,879 participants (predominantly males [78.5–95.3%], aged 55–66 years). Eligible studies focused on adults with gout and examined the lifestyle or dietary factors affecting uric acid levels or gout activity. The review followed a pre-specified protocol (PROSPERO registration CRD42024594359). To optimize the quality, the bias risk was assessed using the Newcastle-Ottawa scale for observational studies and the Cochrane Risk of Bias 1.0 tool for randomized controlled trials.

**Results:**

The findings suggest that consuming polyunsaturated fatty acid-rich fish, regular physical activity, and increased vegetable intake may reduce gout flares. Conversely, high purine intake (especially animal sources), excessive alcohol consumption, and obesity are risk factors for gout exacerbation. Some studies have reported reduced serum uric acid levels with dietary changes, whereas others have found no significant effect. Despite the variability and recall bias, dietary and lifestyle modifications may help manage gout and reduce disease activity.

**Conclusion:**

The findings of this systematic review emphasize the importance of dietary and lifestyle factors in managing serum uric acid levels and reducing the risk of gout flares. Further research is required to establish clinical recommendations to improve patient outcomes.

**Supplementary Information:**

The online version contains supplementary material available at 10.1186/s41043-025-00982-4.

## Key-points

This systematic review emphasizes the importance of dietary and lifestyle factors in managing serum uric acid levels and reducing the risk of gout flares.

The study findings can be applied in clinical practice to develop more refined guidelines for the treatment and management of gout, helping patients understand the importance of avoiding purine-rich food, alcohol consumption, and sugar-sweetened beverages.

It also emphasizes the importance of optimal body weight and regular physical activities.

**Supplementary Information:**

The online version contains supplementary material available at 10.1186/s41043-025-00982-4.

## Introduction

Gout has historically been known as the “disease of kings” or “king of diseases” because of its acknowledged presence throughout history. It was first reported by Hippocrates in ancient Greece [1, 2]. It is a common and debilitating form of inflammatory arthritis caused by the deposition of monosodium urate (MSU) crystals in the joints [1]. Recurrent flares are described as recurrent attacks of acute inflammatory arthritis. It is characterized by the involvement of the first big toe and the large distal joints of the lower extremities [2, 3]. These flares indicate chronic gout or poor adherence to gout management. Unfortunately, recurrent flares are believed to be among the most excruciating episodes experienced by individuals [3]. Due to its severe symptoms and related joint swelling, gout significantly affects quality of life. An observational study reported that gout has a significant impact on disability and overall quality of life [4]. The pathogenesis of gout involves a complex interplay among genetic, metabolic, and environmental factors. Hyperuricemia is the main feature of gout and results from either overproduction or underexcretion of uric acid [5]. In healthy individuals, uric acid is efficiently eliminated by the kidneys. However, this balance is disrupted in patients with gout, leading to the accumulation of uric acid in the blood. Although hyperuricemia is a primary risk factor for gout, not all patients with elevated uric acid levels will develop gout, highlighting its complexity [6, 7].

Approximately 36% of individuals with hyperuricemia develop gout, and as many as 76% of individuals with asymptomatic hyperuricemia may not have MSU crystal deposition [8, 9]. Other studies have shown that only 17–42% of patients with gout with a serum urate concentration > 8 mg/dL meet the ultrasonographic features of MSU [7]. Hyperuricemia is defined as serum uric acid levels exceeding 6.0 mg/dL in women, 7.0 mg/dL in men, and 5.5 mg/dL in children and adolescents. It is an independent risk factor for gout. Genetics also play a significant role in serum uric acid levels, with 43 genes identified to date that influence it [10]. The rate balance in the body is maintained by a combination of urate production and elimination. Uric acid levels tend to be relatively high in humans and some primates. This is largely due to several inactivating mutations in uricase, which breaks down uric acid, as well as mutations in urate transporter 1 (*URAT1)* that enhance its ability to reabsorb uric acid [11]. Recently, the prevalence of gout has increased. Current estimates suggest that gout affects approximately < 1–6.8% of individuals globally. Gout is more common in men and older individuals [12].

Provided that patients meet certain fit-for-treatment criteria, urate-lowering therapy (ULT) is prescribed for gout after the initial diagnosis. According to current treatment practices described by Ragab et al. [2] and Dehlin et al. [12], ULT aims to maintain serum urate levels below saturation thresholds to prevent flares and joint damage. The mechanism of ULT can be ultimately summarized through two categories: drugs that decrease purine breakdown and drugs that increase the urinary elimination of established uric acid, which are xanthine oxidase inhibitors (XOIs) and uricosuric medications, respectively [12]. According to Aitken (2017), as cited in Dehlin et al. [12], the first XOI was allopurinol; febuxostat later replaced it due to greater potency and easier dosing, despite higher cost and cardiovascular concerns. On the other hand, there are multiple uricosuric agents. Namely, probenecid, benzbromarone, sulfinpyrazone, and lesinurad are difficult to use in patients with severe chronic kidney disease and a high propensity to form kidney stones, in addition to their demanding dosing regimen, as reported by Saag et al., Dalbeth et al., and Aitken (all cited in Dehlin et al. [12]).

Despite the established treatment guidelines, gout remains poorly managed in most cases. Furthermore, ULT may not be initiated promptly after the diagnosis by physicians [13]. In recent years, lifestyle factors, such as diet, physical activity, and body weight as described by the body mass index (BMI), have gained significant attention in the treatment of gout. In an observational study, Rees et al. reported that when patients were provided lifestyle advice and education along with ULT, 92% were able to achieve their therapeutic targets [14]. Alterations in uric acid metabolism are associated with certain dietary patterns, particularly the consumption of food high in purine, alcohol, and fructose-sweetened beverages. These lifestyle and dietary patterns not only influence disease activity but also play a pivotal role in the initial development of gout, as they directly contribute to sustained hyperuricemia and urate crystal formation [13–15]. Purine-rich foods such as red meat and peans contribute to increased uric acid production. A retrospective study from China reported that hyperuricemia increased by 2.40% with each 10 g intake of animal-derived food sources [15]. The effect of specific individual dietary patterns on gout has been extensively studied and associated with hyperuricemia and gout risk, but comprehensive analyses of overall dietary patterns and lifestyle factors remain limited. This systematic review aims to address the current evidence on the combined impact of dietary patterns and lifestyle factors on serum uric acid levels and gout activity, thereby addressing existing gaps and providing a more holistic understanding of modifiable risk factors.

## Methods

This systematic review was conducted in accordance with the Preferred Reporting Items for Systematic Reviews and Meta-Analyses (PRISMA) guidelines [16]. The PICOS structure for this review was as follows: Patients (P), adult patients with gout; intervention (I), lifestyle factors and dietary patterns; comparison (C), standard care or no intervention; outcomes (O), serum uric acid levels and frequency of gout flares; study design (S), randomized controlled trials and cohort studies.

### Search strategy and data sources

A systematic search was performed using several databases to identify relevant studies on the impact of lifestyle factors and dietary patterns on serum uric acid levels and disease activity in gout. Databases such as PubMed, BMJ journals, Google Scholar, the Cochrane Library, and Web of Science were searched for relevant studies. All articles were included up to June 2024. The keywords used for the search included “gouty arthritis,” “gout,” “serum uric acid,” “gout attacks,” and “dietary patterns.” The keywords were combined using Boolean operators “OR” and “AND.” Both the MeSH terms and titles/abstracts were used in the search. The inclusion criteria of this systematic review were as follows: (1) studies focusing on adults with gout; (2) studies examining the impact of lifestyle factors and dietary patterns on serum uric acid levels and disease activity; (3) studies providing specific data on lifestyle factors, dietary patterns, and disease outcomes; (4) cohort and randomized controlled trials; and (5) studies published in English. The exclusion criteria were as follows: (1) studies not addressing lifestyle or dietary factors; (2) animal studies, non-human studies, or experiments; (3) editorials, letters, commentaries, case reports, case series, and review articles; and (4) studies published in languages other than English were excluded to ensure accurate data extraction and interpretation; however, we acknowledge that this may introduce language bias.

### Data extraction

After retrieving the database search, the results were merged by the reference manager (EndNote^®^ X7). The file was then transferred to Rayyan (Rayyan Systems Inc., MA, USA), a software designed for screening studies [17]. Before screening, duplicates were removed from the studies. Two independent reviewers were blinded to each other’s selection of studies and involved in the screening process. First, the studies were screened based on their titles and abstracts. After the first step, blinding was removed, and conflicts were resolved after discussion. A third reviewer was involved in any disagreement regarding the finalization of the studies, and the decision was made based on the majority’s opinion. Full-length screening was performed, and the key findings and demographic details of the participants were recorded in an Excel file. The included studies were conducted across multiple countries, specifically the United States (three studies), South Korea, Australia, Norway (two studies), and the United Kingdom, reflecting a diverse geographical distribution of research.

### Risk of bias assessment

The risk of bias was assessed by two authors. The Cochrane Risk of Bias 1.0 tool (ROB 1.0) was used to assess the methodological quality of the included randomized controlled trials and crossover studies, and the Newcastle-Ottawa Scale was used for observational studies.

### Outcomes

The outcome variables investigated in this systematic review included lifestyle factors (physical activity levels, alcohol consumption patterns, and smoking status), dietary patterns (high-purine food intake, fructose consumption, and overall diet quality), and disease activity measures (serum uric acid levels, frequency of gout flares, and joint involvement).

### Data synthesis

Due to the heterogeneity in interventions, outcome measures, and study designs among the included studies, a meta-analysis was not feasible. Therefore, a narrative synthesis approach was used to summarize and interpret the findings. The synthesis was structured to cover dietary patterns, lifestyle modifications, and their respective impacts on serum uric acid levels and gout activity. Results were organized in tables based on the included studies. Quantitative data from included studies were presented where available, including means, medians, odds ratios (ORs), and confidence intervals (CIs).

## Results

### Included studies

The literature search provided an overall count of 173 studies: PubMed (*n* = 5), BMJ Journals (*n* = 17), and Google Scholar (*n* = 151). Searches on Web of Science and Cochrane did not yield any results. Before screening, eight duplicates were removed, yielding 165 full-text studies that were screened. During the screening, 121 records were removed based on the title and abstract. The remaining 44 studies were retrieved, of which three articles were not found. After evaluating 41 papers for eligibility, only eight articles were included in this systematic review and meta-analysis.

### Flow diagram

**Fig. 1:**
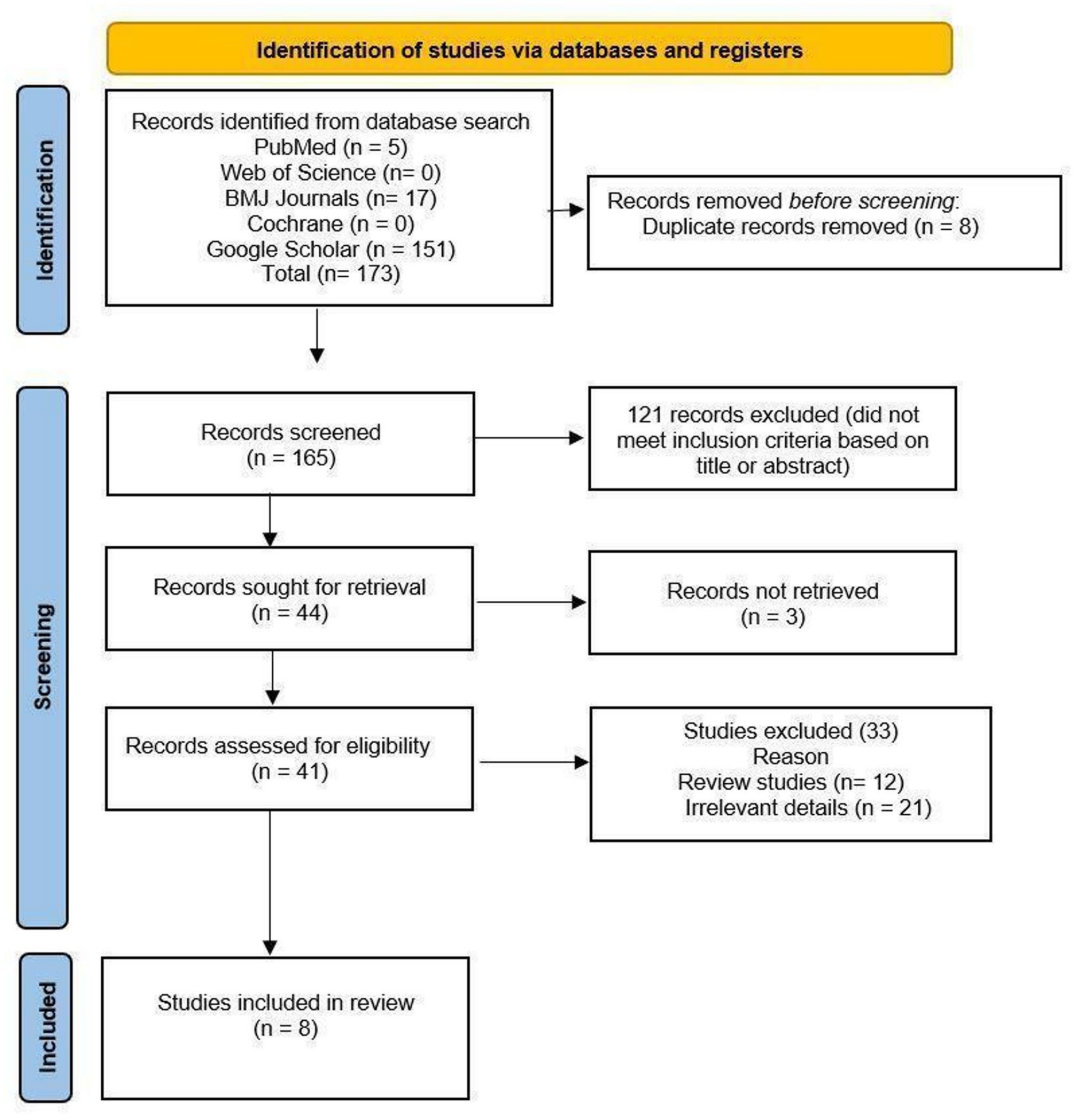
PRISMA flow diagram. PRISMA, Preferred Reporting Items for Systematic Reviews and Meta-Analyses

Figure 1 shows the PRISMA flow diagram for this systematic review.

### Study characteristics

Table 1 presents the detailed characteristics of the included studies. In total, 47,879 participants were included in the studies. Most of the studies were prospective cohort studies (*n* = 5), with two case-crossover studies and one prospective randomized controlled trial. Most studies had a mean participant age of 55–66 years. The proportion of male sex was 78.5–95.3%. Dietary interventions varied, including n-3 polyunsaturated fatty acid (PUFA) supplements, purine-rich foods, and Dietary Approaches to Stop Hypertension (DASH) diet.

**Table 1:** Summary of the included studies

Sr. No.	Author, Year	Study design	Total participants (*n*)	Age (Years)	Sex, Male (%)	BMI	Disease history	Dietary regimen	Physical activity
1	Zhang et al. [18] 2019	Internet-based, case-crossover study	724	54.5 ± 12.5	78.5	32.1 ± 6.9	Gout diagnosis and a gout flare within the last 12 months	n-3 PUFA-rich supplements (fish oil, n-3 PUFA supplements, and cod liver oil) as one intervention and n-3 PUFA-rich fish as another intervention	N/A
2	Do et al. [19] 2024	Prospective cohort (multi-center study)	232	55.08 ± 17.69	90.5	26.47 ± 3.99	Acute gout flare (more than twice in the past year in 79.8% of patients)	Alcohol, coffee, soft drinks, meat, dairy, vegetables, fruit, and exercise	N/A
3	Holland et al. [20] 2014	Prospective, randomized controlled trial	30	64 (44–80)	93.1	29 (23–35)	Intervention group: duration of gout, median (range) (years), 5 (1–40); flares during last 6 months, median (range), 0 (0–4) Control group: duration of gout, median (range) (years), 10 (1–49); flares during last 6 months, median (range), 2 (0–5)	Dietary advice recommended reducing red meat intake, avoiding offal, shellfish, and yeast extract; and including low fat dairy products, vegetables, cherries, and the potential benefit of coffee and vitamin C	N/A
4	Zhang et al. [21] 2012	Case cross-over	633	N/A	N/A	30.6 (14.7–69.9)	Years of disease duration: 5 (0–49)	Purine-rich food	N/A
5	Uhlig et al. [22] 2023	Prospective observational study	211	56.4 ± 13.7	95.3	28.8 ± 4.5	Years of disease duration: 7.8 ± 7.6	Sugar-sweetened soft drink	Physical activity at least three times weekly: 30.4%
6	Watson et al. [23] 2022	Prospective cohort study	1,184	66.61 ± 12.4	83.6	29.13 ± 5.11	Years of disease duration: 11.91 ± 12.13; occurrence of current flare: 132 ± 11.1	DASH Diet	N/A
7	McCormick et al. [3] 2020	Prospective cohort study	44,654	54 ± 9.8	N/A	N/A	Nil	DASH Diet	N/A
8	Uhlig et al. [24] 2021	Prospective cohort study	211	56.4 ± 13.7	95.3	28.8 ± 4.5	Years of disease duration: 7.8 ± 7.6	Alcohol, smoking, and sugar-sweetened drinks consumed daily	Physical activity at least three times weekly: 30.4%

DASH, Dietary Approaches to Stop Hypertension; PUFA, polyunsaturated fatty acids; BMI, body mass index; N/A, not applicable

Table 2 provides descriptive details of the included studies. Various protective and risk factors for gout have been identified. Protective factors against gout include dietary consumption of n-3 PUFA-rich fish, regular exercise, and increased vegetable consumption. The notable risk factors reported in these studies include purine intake, high waist circumference (WC), low-density lipid cholesterol (LDL-C) levels, excess adiposity, and alcohol consumption. Some of these risk factors were assessed in relation to the gout impact scale (GIS) score or health-related quality of life (HRQOL) in some studies. Only four studies reported serum uric acid levels. Only two studies reported a significant difference in serum uric acid levels [18, 19], whereas two studies did not report any significant difference in serum uric acid levels [20, 21].

**Table 2:** Follow-up duration and outcome properties

Author, Year	Baseline serum uric acid level	Urate levels post-intervention	Key findings	Conclusion
Zhang et al. [18] 2019	N/A	N/A	- Among 724 participants, 22% reported consuming n-3 PUFAs in the prior 48 h. - n-3 PUFA supplements did not significantly reduce the risk of recurrent gout flares. - Consumption of fatty fish was associated with a 33% lower risk. - A dose-response effect was observed, with greater reduction in flare risk for those consuming two or more servings (OR = 0.74 [95% CI: 0.54–0.99]).	After adjusting for total purine intake, dietary consumption of n-3 PUFA-rich fish was associated with a reduced risk of recurrent gout flares.
Do et al. [19] 2024	8.63 ± 1.90 mg/dL	N/A	- Patients who exercised more frequently and consumed less meat and soft drinks had significantly lower overall gout concern scores. - Those who consumed vegetables and exercised more frequently reported lower well-being during gout attacks (*p* = 0.04 and *p* = 0.01, respectively). - A negative linear relationship was identified between vegetable consumption and overall GIS score, as reflected in the scale’s subcategories such as “well-being during attacks” and “gout-related concerns during attacks”.	Although a negative correlation was observed between GIS scores and both vegetable consumption and exercise, the overall findings of the study support the hypothesis that increased vegetable intake and regular exercise are associated with improved quality of life and healthier lifestyles.
Holland et al. [20] 2014	Intervention: 0.29 ± 0.08 mmol/L Control: 0.29 ± 0.08 mmol/L	At 6 months: Intervention: 0.30 ± 0.08 mmol/L Control: 0.27 ± 0.07 mmol/L	- No statistically significant improvement was observed in the urate levels at 6 months follow-up after intervention. - Treatment group showed a significant increase in knowledge level at follow-up (*p* < 0.05).	Nutritional education does not result in significant improvement in clinical parameters related to gout.
Zhang et al. [21] 2012	N/A	N/A	- Higher total purine intake over 2 days significantly increased the odds of recurrent gout attacks (from OR = 1.17, 95% CI: 0.88–1.55 to OR = 4.76, 95% CI: 3.37–6.74) (*p* < 0.001). - Animal-source purines showed stronger associations, with OR ranging from 1.42 (95% CI: 1.07–1.87) to 2.41 (95% CI: 1.72–3.36) (*p* < 0.001). - Plant-source purines had weaker associations, with OR between 1.12 (95% CI: 0.84–1.49) and 1.39 (95% CI: 0.98–1.98) (*p* = 0.04).	Purine intake increases the risk of gout by fivefold. The risk is more severe with animal sources.
Uhlig et al. [22] 2023	500 ± 77 µmol/L	2 years: 324 ± 70 µmol/L	- Higher LDL-C levels were associated with an increased risk of gout flares during the second year (OR = 1.8, 95% CI: 1.2–2.6). - A lower baseline waist circumference increased the likelihood of achieving serum uric acid targets.	High WC and lipid levels were associated with adverse gout outcomes.
Watson et al. [23] 2022	44.13 ± 2.52 mg/dL	6 months: 43.86 mg/dL 12 months: 43.92 mg/dL 24 months: 43.74 mg/dL 36 months: 43.74 mg/dL	- Patients experiencing frequent flares were more likely to be socioeconomically deprived, obese, have chronic kidney disease, and present with more severe flare patterns. - Factors linked to worse HRQOL included severe pain, polyarticular flares, depression, and allopurinol use.	Frequent and severe gout flares were associated with poor clinical and quality-of-life outcomes.
McCormick et al. [3] 2020	N/A	N/A	- In men, 77% of confirmed gout cases could potentially be prevented if they maintained a normal weight and followed a diet akin to the DASH. - Excess Adiposity was a major modifiable risk factor contributing to gout incidence.	Addressing excess adiposity can prevent the risk of gout.
Uhlig et al. [24] 2021	500 ± 77 µmol/L	Month 12: 311 ± 48 µmol/L	- Mean serum uric acid levels significantly decreased from 500 to 311 µmol/L, with 85.5% of patients reaching the treatment target. - Factors associated with lower chances of achieving the target included weekly alcohol consumption (OR = 0.14; 95% CI: 0.04–0.55). - Beliefs in overuse of medications were also linked to lower treatment success (OR = 0.77; 95% CI: 0.62–0.94).	Decreased consumption of alcohol and higher self-efficacy are associated with treatment success in gout.

HRQOL, health-related quality of life; OR, odds ratio; PUFA, polyunsaturated fatty acid; WC, waist circumference; GIS, gout impact scale; N/A, not applicable; LDL-C, low-density lipid cholesterol

**Fig. 2:**
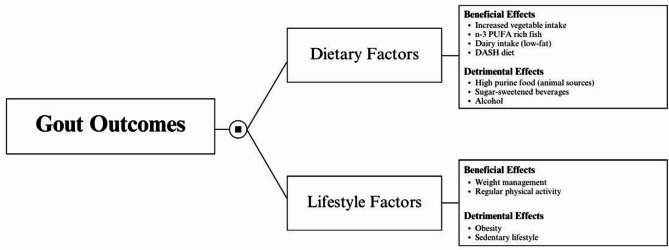
A conceptual figure summarizing variables and their effect on gout. PUFA, polyunsaturated fatty acid; DASH, Dietary Approaches to Stop Hypertension

Figure 2 portrays a summary of the relationship between the study variables and their effect on gout management. It includes both variables that had a positive impact and those that exerted a negative effect.

### Quality assessment of the included studies

Apart from one study that had a moderate risk of bias [22], all studies had a low risk of bias (Table 3). Follow-up was inadequate in four studies, and two studies did not have a sufficient follow-up duration. Only one study had an inadequate selection of non-exposed cohorts.

**Table 3:** Risk of bias measured with the Newcastle-Ottawa scale

	Selection				Comparability		Outcome			
Study	Representativeness of the exposed cohort	Selection of the non-exposed cohort	Ascertainment of exposure	Demonstration that outcome of interest was not present at the start of study	Controls for the most important risk factors	Controls for other risk factors	Assessment of outcome	Followed up long enough for outcomes to occur	Adequacy of follow-up of cohorts	Total quality score
**Do et al.** [19]	1	0	1	1	1	1	1	0	0	6
**Uhlig et al.** [22]	1	1	1	1	1	1	1	1	0	8
**Watson et al.** [23]	1	1	1	1	0	1	1	1	1	8
**McCormick et al.** [3]	1	1	1	1	1	1	1	0	0	7
**Uhlig et al.** [24]	1	1	1	1	1	1	1	1	0	8

**Fig. 3:**
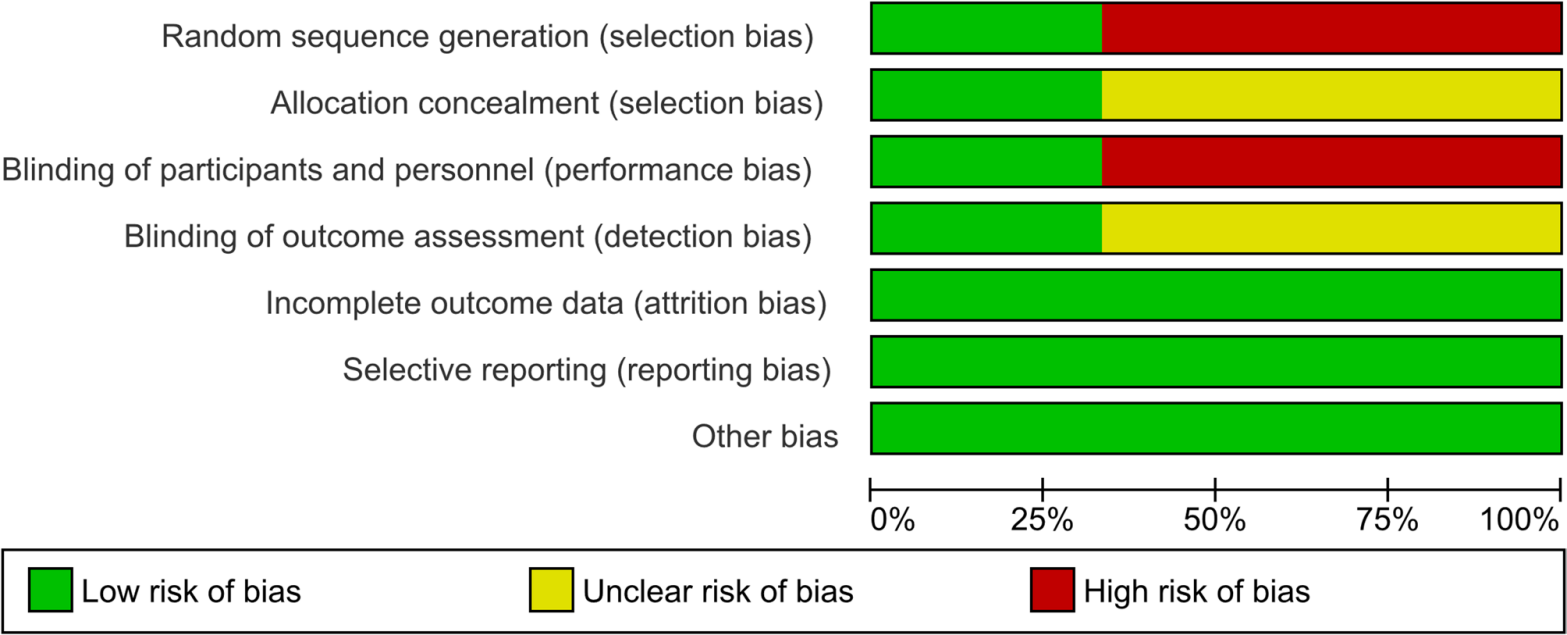
Risk of bias graph

**Fig. 4:**
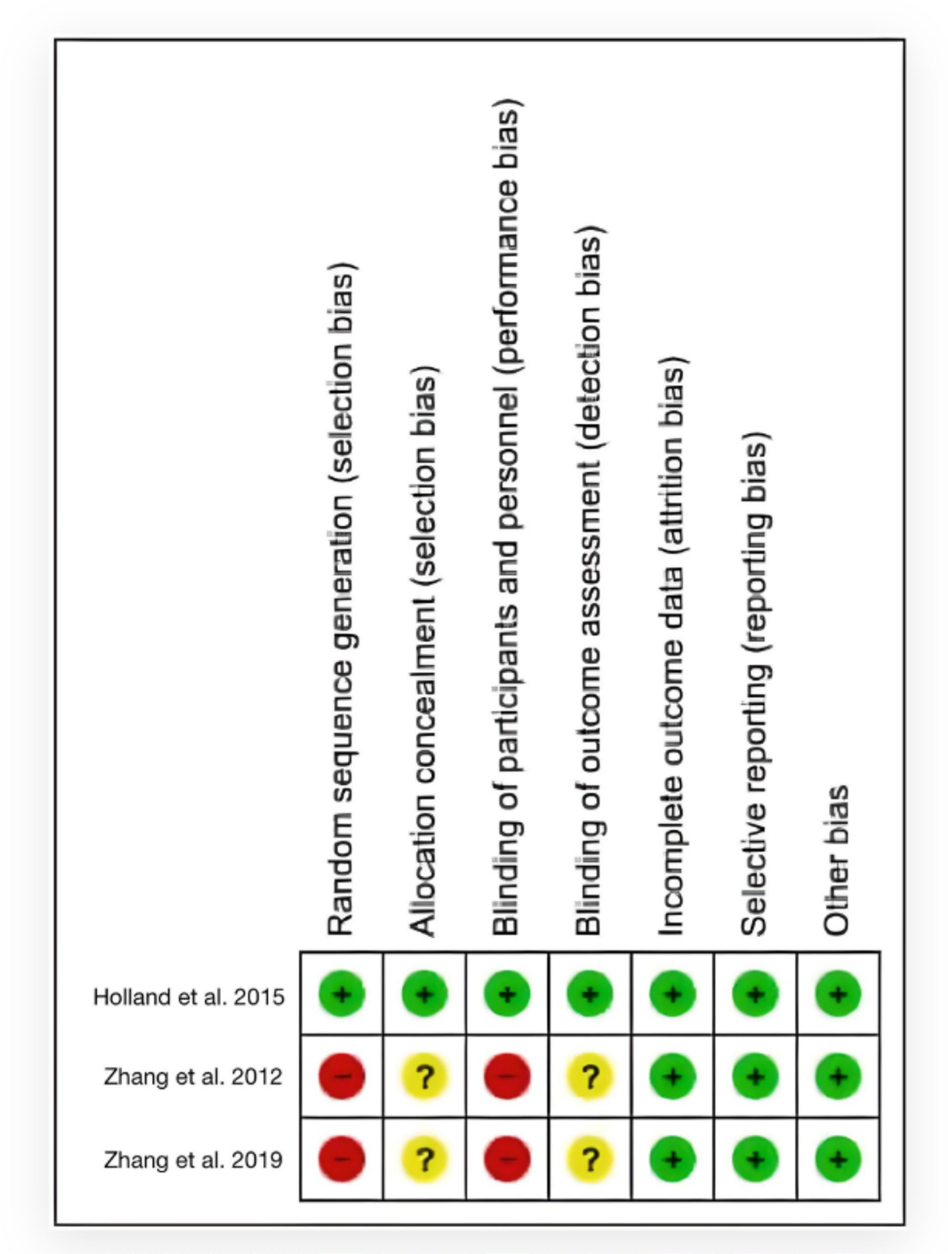
Risk of bias summary

Figure 3 shows the risk of bias in the included studies. Of the three studies, selection bias was reported in two. Similarly, performance bias was observed in two studies. Selection and detection biases were unclear in two studies. All other biases (attrition, reporting, etc.) were low in the included studies. (Figures 3 and 4).

## Discussion

### Overview of findings

This systematic review demonstrated that dietary and lifestyle factors have a remarkable impact on serum uric acid levels and the management of gout symptoms. Several risk factors from the included studies included purine intake (especially from animal sources), fructose-sweetened beverages, alcohol consumption, high waist circumference, and elevated lipid levels. These factors contribute to recurrent gout flares and increased serum uric acid levels. Similarly, the protective effects of n-3 PUFA-rich fish, regular exercise, and increased vegetable consumption have been documented. However, some interventions showed limited or no improvement in clinical outcomes. Holland et al. reported no significant effect of nutritional education on serum urate levels after 6 months. However, with the educational interventions, knowledge significantly increased among patients [20]. A previous systematic review and meta-analysis by Ramsubeik et al. reported that patients who received education or behavioral interventions were more likely to achieve the desired serum urate level of < 6 mg/dL than those who did not receive an intervention (47.2% vs. 23.8%) [23].

#### Impact of dietary interventions

The effects of dietary interventions on gout management are well recognized in the literature. Our findings are consistent with those of a prior systematic review that investigated the role of dietary intervention on gout and cardiovascular risk factors; Vedder et al. included 18 studies in their systematic review. They showed that low-calorie, low-purine, and DASH diets significantly reduced the risk of gout [24]. Similarly, another systematic review and meta-analysis showed that red meat, alcohol, and seafood consumption were positively associated with gout and hyperuricemia. The authors also showed that dairy products and soy foods were negatively associated with gout. Furthermore, coffee intake was negatively associated with gout; however, it was positively associated with hyperuricemia in women, but not in men [25]. This systematic review revealed that certain dietary patterns and food choices can significantly influence serum uric acid levels and the frequency of gout flares. For instance, diets rich in n-3 PUFAs from fatty fish have been associated with a reduced risk of recurrent gout flares [26]. Zeng et al. found no correlation between an n-3 PUFA-rich diet and gout prevention in their study. Nonetheless, they identified that an n-3 PUFA-poor diet increased the risk of gout by 8.7% [27].

This paper also showed that increased intake of purine-rich foods, particularly from animal sources, was consistently linked to higher serum uric acid levels and an increased risk of gout attacks [28]. Food rich in purines has always been implicated in the development of gout and increased uric acid levels [29]. Additionally, our study also highlights that the consumption of sugar-sweetened beverages is associated with higher uric acid levels and an increased risk of gout flares [22]. Similar to our results, a systematic review and meta-analysis showed that fruit juices (relative risk [RR] = 1.77, 95% CI: 1.20–2.61) and sugar-sweetened beverages had an exacerbating trend with gout (RR = 2.08, 95% CI: 1.40–3.08). In spite of this, fruit intake was not associated with gout [30]. Similarly, another systematic review and meta-analysis confirmed a positive correlation between sugar-sweetened beverage consumption and risk of gout development. One meta-analysis reported that such beverages increase the risk by 35% [31]. Furthermore, Lee et al., in their meta-analysis of three studies involving 2,606 patients with gout, concluded that sugar-sweetened beverages were significantly associated with the risk of gout (RR = 1.98, 95% CI: 1.447–2.725) [32].

#### Role of lifestyle factors

A significant part of gout management involves physical activity and weight management, as regular exercise was found to be influential in reducing “gout concern” scores and the overall well-being of individuals [22]. Shah et al. reported similar findings: their study revealed a significant reduction in gout flares in physically active patients [33]. Choi et al. also concluded that regular exercise can decrease the risk of gout [34]. Healthy weight maintenance has also been presented as a preventive measure for gout; likewise, obesity is suggested to be associated with unfavorable gout outcomes [3]. In a meta-analysis by Aune et al., it was reported that the overall RR with regard to gout for a 5-unit increase in body mass index (BMI) was 1.55 (95% Cl: 1.44–1.66) [35]. Evans et al. described that the likelihood of developing gout increases 2.24 times in individuals with a BMI ≥ 30 kg/m^2^ (95% CI: 1.76–2.86) [36]. Similarly, a systematic review encompassing ten studies was conducted, with the majority of studies concluding that weight loss decreases the risk of gout [37].

#### Biological association between dietary factors and gout outcomes

Sustained hyperuricemia is the hallmark of gout pathophysiology, which results from increased production or reduced excretion of uric acid. Some previous studies discussed the association between dietary factors and gout outcomes. High purine intake can increase the level of purine metabolism [2, 6, 15]. In addition, fructose-containing beverages, including sugar-sweetened sodas, promote uric acid production by enhancing adenosine triphosphate (ATP) breakdown and elevated purine nucleotide turnover [30, 31]. Conversely, some studies found that some diets have a protective effect in gout through lower inflammation and reduced flare frequency, such as plant-based diets in vegetables, fibre, polyphenols, and dairy products [2, 22, 24]. Omega-3 polyunsaturated fatty acids, known for their anti-inflammatory properties, have shown promise in reducing recurrent gout flares [6]. Furthermore, vitamin C may reduce oxidative stress and inflammation, which are the hallmarks of the pathogenesis of gout flares [2]. In conclusion, these dietary factors in association with crystal deposition, inflammation, and immune response play a significant biological role in clinical gout [6]. The dietary interventions underscore the role of lifestyle modification in the prevention and management of gout.

### Strengths and limitations

This systematic review has several strengths and limitations that should be considered when interpreting the findings. The main strength of this study was the systematic search of various databases to identify relevant studies. This study has some limitations that should be acknowledged. The main limitation is the notable heterogeneity in the dietary interventions and outcome measures used across the studies. For example, post-intervention serum uric acid levels were not reported in 50% of studies. Similarly, different dietary interventions were used in the studies, with some focusing on n-3 PUFA supplements and others examining broader dietary patterns (such as the DASH diet), thereby limiting the generalizability of the findings. Furthermore, most studies had a predominance of male participants because of the nature of the disease. Most studies did not account for confounding factors such as medication use, socioeconomic status, and comorbidities in their analysis. Another possible limitation is publication bias, as studies showing statistically significant positive findings are more likely to be published than those with negative outcomes, potentially distorting the results of this study. Additionally, the review process has its own constraints. The absence of specific outcome data prevented the conduct of a meta-analysis to assess the impact of a particular intervention on gout.

### Implications for practice and research

The findings of this systematic review emphasize the importance of dietary and lifestyle factors in managing serum uric acid levels and reducing the risk of gout flares. These findings can be applied in clinical practice to develop more refined guidelines for the treatment and management of gout, helping patients understand the importance of avoiding purine-rich food, alcohol consumption, and sugar-sweetened beverages. Additionally, patients with gout should aim for optimal body weight, with regular physical activity. Nonetheless, gaps in the current research highlight the need for further investigation, focusing on the long-term effects of certain dietary factors on uric acid levels and gout management. Additional randomized controlled trials are needed to establish solid causal relationships and to identify optimal dietary and lifestyle interventions for gout management. Based on these findings and the overall strengths of the review, it is recommended that future guidelines incorporate structured lifestyle counseling addressing both nutrition and exercise as a key component of gout management [23, 33]. Furthermore, public health strategies should consider broader educational campaigns targeting modifiable risk factors to reduce the burden of gout [23, 34]. Lastly, this study sheds light on the importance of lifestyle and behavioral interventions in the treatment of chronic gout, which could also be the case with other chronic conditions; therefore, it is essential to highlight the need for future studies to adhere to evolving data standards in patient data governance and participant privacy. Emerging standards, such as that proposed by Zhang et al., emphasize the need for a transparent consent process with data anonymization and appropriate safeguards based on the sensitivity of collected information. While our review did not involve primary data collection, future cohort and interventional studies should integrate this framework to ensure patients’ trust and privacy as well as responsible data sharing [38].

## Conclusion

The findings of this systematic review emphasize the importance of a holistic approach to gout management that encompasses both pharmacological and non-pharmacological interventions. The key dietary factors identified included the beneficial effects of n-3 PUFA fishes and increased vegetable intake, in contrast to the detrimental effects of purine-rich foods (especially animal sources), sugar-sweetened beverages, and alcohol consumption. Lifestyle factors, particularly weight management, have emerged as crucial elements for controlling gout symptoms and improving the overall quality of life. These findings have important implications for clinical practice, suggesting that healthcare providers should integrate comprehensive dietary counseling and lifestyle recommendations into standard gout management protocols, such as increasing the intake of oily fish to 2–3 times per week, limiting the consumption of red meats, sugar-sweetened beverages, and alcohol, and encouraging regular weight monitoring and weight loss when appropriate. Future research should focus on developing personalized dietary and lifestyle interventions that consider individual patient factors and comorbidities.

## Electronic supplementary material

Below is the link to the electronic supplementary material.

Supplementary Material 1

Supplementary Material 2

## Data Availability

No datasets were generated or analysed during the current study.

## Declarations

**of Generative AI and AI-assisted technologies in the writing process**.

During the preparation of this work, the authors used ChatGPT solely for proofreading purposes. After using ChatGPT, the authors reviewed and edited the content as needed and take full responsibility for the content of the publication.

## List of Abbreviations

MSU: Monosodium urate
URAT1: Urate transporter 1
ULT: Urate-lowering therapy
XOIs: Xanthine oxidase inhibitors
MDs: Mean differences
CIs: Confidence intervals
IQRs: Interquartile ranges
SD: Standard deviation
RRs: Risk ratios
RR: Relative risk
ORs: Odds ratios
WC: Waist circumference
LDL-C: Low-density lipid cholesterol levels
N/A: Not applicable
BMI: Body mass index
GIS: Gout Impact Scale
HRQOL: Health-related quality of life
n-3 PUFA: n-3 polyunsaturated fatty acid
DASH: Dietary Approaches to Stop Hypertension
ATP: Adenosine triphosphate
