# Supplementary material for: Impact of lifestyle factors and dietary patterns on serum uric acid levels and disease activity in gout: a systematic review
Source: J Health Popul Nutr. 2025 Jul 2;44:223. doi: 10.1186/s41043-025-00982-4 (PMC12225473; doi:10.1186/s41043-025-00982-4)
Supplement: Supplementary file 2 — Supplementary Material 2 [file 41043_2025_982_MOESM2_ESM.pdf]

Authorship and Disclosure Form

Manuscript ID number (if known): ??

Article title (first few words): Impact of Lifestyle Factors and Dietary Patterns on Serum Uric Acid Levels and Disease Activity in Gout: A Systematic Review

Corresponding author: Mohammad Mustafa

Email address: Mamustafa@uj.edu.sa

Full list of co-authors: Shahad Alshamrani, Lama Alghamdi, Hala Danish, Dana Alamoudi, Ghala Alshamrani, Abdullah Alagha, Adnan Alshaikh, Saher Alqarni, Yassir Bawazir

Please note: It remains the responsibility of the corresponding author to ensure all co-authors are named here, and within the manuscript.

COPYR IGHT:
The article is published under the Creative Commons Attribution-Noncommercial License (http://creativecommons.org/licenses/by-nc/4.0) which allows users to read, copy, distribute, and make derivative works for non-commercial purposes from the material, as long as the author of the original work is cited.

The author assigns the exclusive right to any commercial use of the article to Springer Healthcare Ltd.

As an author of this article, I certify that none of the material in the manuscript (including tables and figures) has been previously published, nor is it included in any other manuscript. If my manuscript includes figures/tables previously published, I certify that I have requested and have been granted permission to reproduce this material.

The author warrants that their contribution is original. The author signs for and accepts responsibility for releasing this material on behalf of any and all co-authors. The assignment covers the exclusive commercial right and license to reproduce, publish, distribute, archive and sell the article in all forms and media of expression now known or developed in the future, including reprints, translations, photographic reproductions, microform, electronic form (offline, online) or any other reproductions of similar nature.

If the article contains, or will contain, digital features (e.g., slide sets, videos, animations, video abstracts, infographics, podcasts, plain language summaries), these are also published under the Creative Commons Attribution-Noncommercial License (outlined above). Such features shall be either hosted on Springer Healthcare Ltd's own website(s) or on a third party website hosted on behalf of Springer Healthcare Ltd. If any features contain figures or tables that have been previously published, I certify that I have requested and have been granted permission to reproduce the material where applicable. Springer Healthcare Ltd. holds exclusive hosting and linking rights to these features.

AUTHOR SHIP:
I, on behalf of all named author(s), certify that:

- I/we have made substantial contributions to the conception or design of the work; or the acquisition, analysis, or interpretation of data for the work; AND
- I/we have drafted the work or revised it critically for important intellectual content; AND I/we give approval of the final submitted version of the manuscript; AND
- I/we agree to be accountable for all aspects of the work in ensuring that questions related to the accuracy or integrity of any part of the work are appropriately investigated and resolved.

Authorship and Disclosure Form

Adis

By signing below, I agree to the following declaration being added to the acknowledgments of the manuscript: "All named authors meet the International Committee of Medical Journal Editors (ICMJE) criteria for authorship for this article, take responsibility for the integrity of the work as a whole, and have given their approval for this version to be published."

I certify that this manuscript is not under consideration for publication elsewhere, nor has it been submitted or accepted in another publication in any form.

Any change in the authors after initial submission must be approved by all authors, and any alterations (additions, deletions or change in corresponding author) must be explained.

For manuscripts that are the report of a study, I confirm that this work is an accurate representation of the trial results.

The rights or interest in the manuscript have not been assigned to any third party.

Moreover, should the editor of the journal request the data upon which the manuscript is based, I shall produce it.

FUNDING DISCLOSURE/ACKNOWLEDGMENTS:
I, on behalf of all named authors, certify that any financial or other conflicting interests such as employment, stock ownership, grants, travel support, royalties, honoraria, paid expert testimony, consultancies, patents (planned, issued or pending), or other (please err on the side of caution), as well as any personal relationships, academic competition, and intellectual passion which may inappropriately influence my/our actions, have been fully disclosed in the Conflict of Interest section of the paper.

All funding sources supporting the work and the Rapid Service Fee, any medical writing and/or medical communications assistance during the preparation of the manuscript, any persons involved indata collection/analysis, and all institutional or corporate affiliations of mine are fully acknowledged within the Acknowledgments section of the paper.

All persons who have made substantial contributions to the work reported in the manuscript (e.g., data collection, data analysis, or writing or editorial assistance) but who do not fulfil the authorship criteria must be named with their specific contributions in the Acknowledgments section. All persons named must give the authors their written permission to be named in the manuscript.

I/we have had full access to the relevant aggregated data and required information to understand and report these research findings.

By signing below, I, on behalf of all named authors, assert that there are no undisclosed conflicts of interest (both personal and institutional) regarding specific financial interests that are relevant to the work conducted or reported in this manuscript that have not been disclosed within this manuscript.

Author's signature

Mohamed Attiyah

Printed name and date

Mohammad Mustafa 13/2/2025

Completed disclosure forms<u>must</u>be uploaded during the online submission process. Completed forms can be submitted in PDF, word document, or image file formats.

Springer

http://www.springer.com/journal
